# Controllable and Diversiform Topological Morphologies of Self‐Assembling Supra‐Amphiphiles with Aggregation‐Induced Emission Characteristics for Mimicking Light‐Harvesting Antenna

**DOI:** 10.1002/advs.202001909

**Published:** 2020-09-23

**Authors:** Shuang Fu, Xiang Su, Meng Li, Shanliang Song, Lei Wang, Dong Wang, Ben Zhong Tang

**Affiliations:** ^1^ Centre for AIE Research Shenzhen Key Laboratory of Polymer Science and Technology Guangdong Research Center for Interfacial Engineering of Functional Materials College of Material Science and Engineering Shenzhen University Shenzhen 518061 P. R. China; ^2^ College of Physics and Optoelectronic Engineering Shenzhen University Shenzhen 518060 China; ^3^ Department of Chemistry Hong Kong Branch of Chinese National Engineering Research Center for Tissue Restoration and Reconstruction The Hong Kong University of Science and Technology Clear Water Bay, Kowloon Hong Kong China

**Keywords:** aggregation‐induced emission, light‐harvesting antenna, supra‐amphiphiles, supramolecular self‐assembly, topological morphologies

## Abstract

Controllable construction of diversiform topological morphologies through supramolecular self‐assembly on the basis of single building block is of vital importance, but still remains a big challenge. Herein, a bola‐type supra‐amphiphile, namely DAdDMA@2*β*‐CD, is rationally designed and successfully prepared by typical host–guest binding *β*‐cyclodextrin units with an aggregation‐induced emission (AIE)‐active scaffold DAdDMA. Self‐assembling investigation reveals that several morphologies of self‐assembled DAdDMA@2*β*‐CD including leaf‐like lamellar structure, nanoribbons, vesicles, nanofibers, helical nanofibers, and toroids, can be straightforwardly fabricated by simply manipulating the self‐assembling solvent proportioning and/or temperature. To the best of knowledge, this presented protocol probably holds the most types of self‐assembling morphology alterations using a single entity. Moreover, the developed leaf‐like lamellar structure performs well in mimicking the light‐harvesting antenna system by incorporating with a Förster resonance energy transfer acceptor, providing up to 94.2% of energy transfer efficiency.

## Graphical abstract

Diversiform topological morphologies including leaf‐like lamella, nanoribbons, vesicles, nanofibers, helical nanofibers, and toroids are straightforwardly realized through supramolecular self‐assembly of a novel aggregation‐induced emission (AIE)‐active supra‐amphiphile under different solvent proportioning and/or temperature. The fabricated supramolecular aggregates perform well in mimicking the light‐harvesting antenna system.

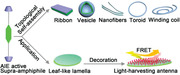

Supramolecular self‐assembly has captivated significant scientific interests not only for the purpose of fundamental studies, but also for performing as functional materials in an extensive range of applications.^[^ ^1^, ^2^, ^3^, ^4^ ^]^ The functions and properties of these self‐assembled aggregates are recognized to be intimately related to their topological morphologies,^[^ ^5^, ^6^, ^7^, ^8^, ^9^, ^10^, ^11^ ^]^ which are undoubtedly lie on molecular arrangements at the supramolecular level.^[^ ^12^, ^13^, ^14^ ^]^ Controlling self‐assembly conditions has been proven to be a powerful protocol to change the intramolecular interactions and molecular packing model, and thus regulate the supramolecular topological structure transformation.^[^ ^15^, ^16^, ^17^, ^18^, ^19^, ^20^, ^21^, ^22^, ^23^ ^]^ Nature has provided infinite examples to illustrate this concept, meanwhile inspired researchers to tailor a variety of artificial building blocks,^[^ ^24^, ^25^, ^26^ ^]^ aiming to construct diversiform supramolecular architectures through tuning self‐assembly conditions. On the other hand, utilizing supra‐amphiphiles as building blocks has become one of the most intensive topics in the area of supramolecular self‐assembly during recent years, by virtues of their facile preparation, dynamic features, richer structure, and potential stimuli‐responsiveness and reversibility comparing with conventional amphiphiles.^[^ ^27^, ^28^ ^]^ Notwithstanding the vital importance of manipulating supramolecular self‐assembly architectures in areas ranging from nanotechnology to biotechnology, precious controlling multiple topological morphologies on the basis of single building block, especially of single supra‐amphiphiles, is still particularly challenging even though enthusiastic efforts have been devoted.

Besides the prominent functions resulting from various topological morphologies, some distinctive and amplified features accompanying by the formation of self‐assembled aggregates are also high desirable for intelligent materials. Given the circumstances, of particular interest is a novel family of luminogens with aggregation‐induced emission (AIE) characteristics. AIE refers to a unique phenomenon that some fluorophores are nonemissive or weakly emissive in the molecularly dissolved state but they emit intensively in aggregates due to the restriction of intramolecular motions (RIM).^[^ ^29^, ^30^, ^31^, ^32^, ^33^ ^]^ Ascribe to their intrinsic advantages including bright emission in aggregates, high photobleaching threshold, and high signal‐to‐noise ratio, AIE luminogens (AIEgens) represent a class of extraordinary alternatives for constructing luminescent supramolecular materials. The utilization of AIEgens not only straightforwardly provides valuable insights in mechanistic understanding of supramolecular chemistry, but also powerfully functionalizes supramolecular materials with efficient fluorescence, making AIEgens‐based supramolecular materials highly useful in sensing, bioimaging, and optoelectronic devices.^[^ ^34^, ^35^, ^36^, ^37^, ^38^, ^39^, ^40^, ^41^, ^42^, ^43^ ^]^ Despite these facts, AIEgens usually exhibit nonplanar conformation showing special packing model upon aggregation, and their intermolecular interactions are relatively complicated, which makes it difficult to control the topological morphology formation and transformation of AIE‐based self‐assembling architectures.^[^ ^34^, ^35^ ^]^

In this contribution, a bola‐type supra‐amphiphile, namely DAdDMA@2*β*‐CD, was rationally designed and successfully synthesized through typical host‐guest binding *β*‐CD units with an AIE‐active scaffold DAdDMA,^[^ ^44^, ^45^, ^46^, ^47^ ^]^ which was prepared by functionalizing 2,5‐dimethoxybenzene‐1,4‐dicarboxaldehyde (DMA) with adamantine (Ad) groups (Figure 4A,B). The self‐assembled DAdDMA@2*β*‐CD can be regulated from leaf‐like lamella structure to nanoribbon, vesicle, nanofiber, helical nanofiber, and toroid by switching of solvent proportioning and/or temperature, thus achieving a successive multiple topological evolution of nanostructures. Meanwhile, by incorporating with Förster resonance energy transfer (FRET) acceptor, the supramolecular architectures with high emission property can also serve as a new platform for mimicking light‐harvesting system.

DAdDMA was designed and synthesized according to the synthetic protocol as illustrated in Scheme S1 in the Supporting Information. Amidation reaction smoothly proceeded by employing amantadine and presynthesized acyl chloride derivative as starting materials, producing intermediate product **2** with the yield of 43%. Then, DAdDMA was successfully obtained through coupling reaction between **2** and 2,5‐dihydroxyterephthalaldehyde. The UV–vis and photoluminescence (PL) spectra of DAdDMA in tetrahydrofuran (THF) solution was further acquired. As shown in Figure S7 (Supporting Information), DAdDMA solution in THF displayed a maximum absorption band at 395 nm, as well as weak emission with maximal intensity locating at 466 nm, while its solid emission peak shifts from 466 to 479 nm (Figure S8, Supporting Information). Moreover, the emission intensity was boosted upon the formation of aggregates with adding water to the solution (**Figure** **1**A,B), definitely demonstrating the AIE characteristics, which were also solidly verified by much higher photoluminescence quantum yield in solid state (6%) than that of dilute THF solution (0.3%). It was observed that the emission intensity reached the maxima when water faction was 60%, and DAdDMA can easily precipitate when water faction is over 60% owing to its high hydrophobic nature, thus resulting in intensity decrease. Compared to the solution state, the red shifted emission in aggregates could be attributed to the increased polarity of the local environment upon raising water faction, resulting from the twisted intramolecular charge transfer (TICT) property of DAdDMA. Indeed, AIE tendency and TICT effect are competitive in determining PL intensity. Nevertheless, the enhanced emission feature of DAdDMA in aggregates reveals a stronger AIE tendency than the TICT effect in this system.

**Figure 1:**
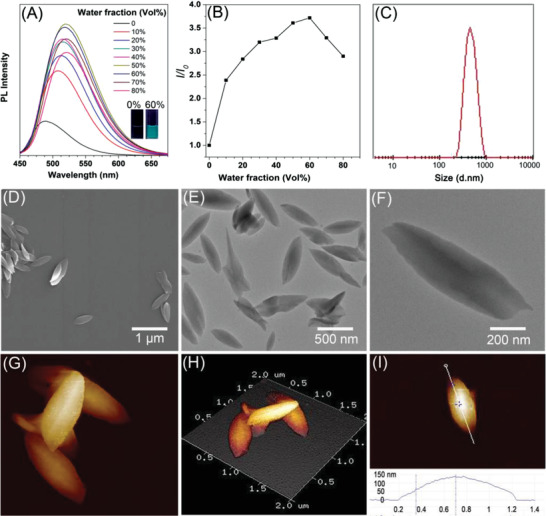
A) Photoluminescence spectra of DAdDMA in THF/water mixtures with different water fractions (insert Photographs taken under UV irradiation emission spectra of DAdDMA at different water fraction). B) The relative fluorescence intensity versus water fraction in the THF/water mixture. C) Size distribution, D) SEM image, E,F) TEM images, and G–I) AFM images of leaf‐like lamellar structure. The samples from Figures 1A–I and 4C were prepared through self‐assembly of DAdDMA@2*β*‐CD in MeCN/H_2_O at 30 °C.

Bola‐type supra‐amphiphile DAdDMA@2*β*‐CD with concentration of 2 × 10^−2^ mol L^−1^ can be straightforwardly generated by mixing 1 equivalent of DAdDMA and 2 equivalents of *β*‐CD in MeCN with the assistance of sonication within 30 min. The typical host–guest binding *β*‐CD units with DAdDMA was demonstrated by ^1^H NMR (Figures S9 and S10, Supporting Information). It was observed that upon slow addition of 500 µL of DAdDMA@2*β*‐CD MeCN solution into 4.5 mL of deionized water under sonication at 30 °C for 60 min, the solution exhibit opalescence, which suggested the formation of supramolecular nanostructures. The critical aggregation concentration (CAC) of DAdDMA@2*β*‐CD was determined to be 0.35 mmol L^−1^ in MeCN/H_2_O mixture (Figure S11, Supporting Information). Dynamic light scattering analysis (DLS) was used to monitor the self‐assembly behavior, and the results showed that the average size of the presented aggregates was ≈479 nm with a restively narrow size distribution (Figure 1C). The morphology of the supramolecular nanostructures was then determined by means of SEM (Scanning Electron Microscope), TEM (Transmission Electron Microscope), and AFM (Atomic Force Microscope). As depicted in Figure 1D–F, SEM image indicated the formation of leaf‐like morphologies with size around 500 nm, and TEM images further confirmed that these aggregates exhibit core–shell lamellar structure. In addition, AFM images revealed that the surface of these leaf‐like lamellar structure with the characteristics of high in the middle and low around (Figure 1G,H), and the height ranges from 10 to 130 nm for the whole micelle (Figure 1I), implying that this new kinds of micelles have big specific surface.

Inspired by the construction of well‐defined nanostructure of self‐assembling DAdDMA@2*β*‐CD, the influence of self‐assembly conditions involving solvent proportioning and temperature were next investigated. It was found that the leaf‐like lamella can be also fabricated by self‐assembly of DAdDMA@2*β*‐CD in other solvents systems with higher polarity, such as DMF (N,N‐Dimethylformamide) /H_2_O (1:9) and EtOH (Ethanol) /H_2_O (1:9). In the DMF/H_2_O mixture, the obtained leaf‐like lamella was curved and aggregated together (**Figure** **2**A) with the average size reaching to 559 nm (Figure S12, Supporting Information), and in the case of EtOH/H_2_O system, the generated leaf‐like lamella became lager (Figure S13, Supporting Information) with the average size reaching to 799 nm (Figure S14, Supporting Information). Furthermore, when the self‐assembly was conducted in mixed THF‐H_2_O solutions with different factions at 30 °C, the topological morphologies of self‐assembled architecture were significantly varied. As illustrated in Figure 2B and Figures S15 and S16 (Supporting Information), huge nanoribbon structure with size distribution from 1000 to 6000 nm was produced by the use of THF/H_2_O (1:9) as solvent; changing the proportion of THF and H_2_O to 2:8 can result in topological transformation from nanoribbons to nanofibers (Figure 2C); while THF/H_2_O ratio was 3:7, left‐handed helical nanofibers was distinctly observed upon self‐assembling of DAdDMA@2*β*‐CD (Figure 2D; Figures S17 and S18, Supporting Information). Circular dichroism (CD) analysis showed that the whole artificial helixes had no CD signal (Figure S19, Supporting Information), suggesting that the artificial helixes could be composed of both left‐handed and right‐handed helical fibers because the DAdDMA@2*β*‐CD is a nonchiral complex. Further increasing the THF faction to 40% caused the partial formation of vesicle structure of self‐assembled aggregates, showing hybrid morphologies of helical nanofiber and vesicle (Figure 2E). Only vesicles were determined when the volume ratio of THF/H_2_O reached to 5:5 (Figure 2F; Figure S20, Supporting Information), and the size of these vesicles was ≈100 nm (Figure S21, Supporting Information). These results evidently demonstrated the multiple morphological evolutions of self‐assembling DAdDMA@2*β*‐CD in the presences of different solvent combinations, and strongly indicated that tiny change of solvent polarity could profoundly trigger the arrangement of building blocks through disturbing the synergistic effect of supramolecular interactions.

**Figure 2:**
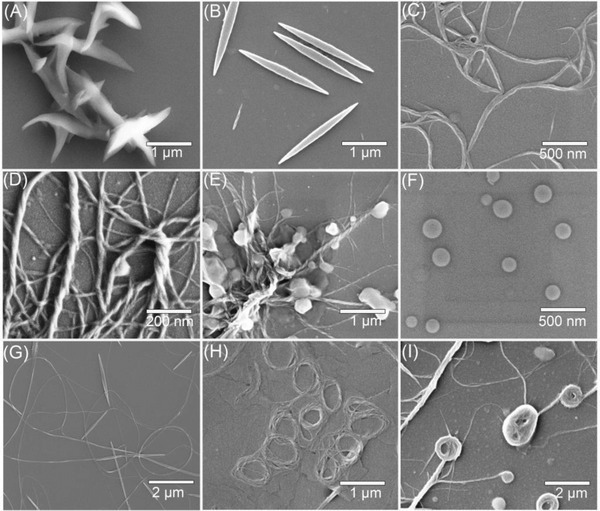
Diversiform topological morphologies of self‐assembling DAdDMA@2*β*‐CD. A) SEM images of leaf‐like lamella for the self‐assembly of DAdDMA@2*β*‐CD in DMF/H_2_O (5:95). B) SEM images of nanoribbons constructed in THF/H_2_O (1:9) at 30 °C. C) SEM images of nanofibers constructed in THF/H_2_O (2:8) at 30 °C. D) SEM images of helical nanofibers constructed in THF/H_2_O (3:7) at 30 °C. E) SEM images of hybrid of helical nanofiber and vesicle constructed in THF/H_2_O (4:6) at 30 °C. F) SEM images of vesicle constructed in THF/H_2_O (5:5) at 30 °C. G) SEM images of hybrid of nanofiber and nanotoroid structures constructed in THF/H_2_O (1:9) at 60 °C. H,I) SEM images of winding coil structures constructed in THF/H_2_O at 60 °C.

Temperature can also regulate the morphology transformation of self‐assembled supra‐amphiphile DAdDMA@2*β*‐CD. As depicted in Figure 2G, by using THF/H_2_O (1:9) system, nanoribbon structure of self‐assembled supra‐amphiphile DAdDMA@2*β*‐CD was directly transformed into nanofibers when the self‐assembly proceeded at 60 °C, and surprisingly, toroid structure was also fabricated concurrently. TEM images revealed that the obtained nanofibers were analogical to that formed in THF/H_2_O mixture with 20% of THF at 30 °C (Figure 2G; Figure S22A,B, Supporting Information), and the average size is ≈1050 nm (Figure S23, Supporting Information). Additionally, these nanofibers tended to bend, even some of them formed toroid structure, which was also clearly determined by TEM analysis (Figure S22C,D, Supporting Information). In the same way, when the other assembly solvent systems (with THF/H_2_O ranges from 2:8 to 4:6) were utilized at 60 °C, nanotoroids can be also generated, but became nanosized superimposed rings just like winding coils (Figure 2H,I; Figures S24 and S25, Supporting Information). To the best of our knowledge, scarcely any of self‐assembled architectures exhibiting nanotoroids and/or winding coils have been previously reported for self‐assembly of AIE molecules. In addition, temperature‐regulated morphological change was also verified by employing MeCN/H_2_O system (Figure 1D; Figure S26, Supporting Information).

Having successfully realized the controllable regulating of topological morphologies of self‐assembling supra‐amphiphile DAdDMA@2*β*‐CD, we tried to further investigate the self‐assembly mechanism. The single crystal structure and molecular stacking patterns of DMA were determined (**Figure** **3**; Table S1, Supporting Information). It was observed that DMA has relatively planar structure in crystal; various inter‐ and intramolecular interactions including weak *π*–*π*, O···H, O···C, and C···H in the crystal lattice, rigidify the molecular conformation and restrict molecular motions. (Figure 3C,D). The planar conformation and abundant interactions of DMA, as well as the supra‐amphiphile features of DAdDMA@2*β*‐CD, enable DAdDMA@2*β*‐CD to effectively self‐attract driven by multiple supramolecular interactions, and to be easily influenced by the self‐assembling environment. Based on the features of the new building block and the diversiform topological morphologies, and according to these previous reports,^[^ ^9^, ^48^, ^49^, ^50^ ^]^ we suggest that all the topological morphologies formations involved hierarchical self‐assembly process (**Figure** **4**C), which was monitored by SEM using MeCN/H_2_O system. As shown in Figure S27A (Supporting Information), small nanoparticles were generated after DAdDMA@2*β*‐CD was dispersed in water for 5 min, and these nanoparticles gradually aggregated into small nanopatches within 15 min (Figure S27B, Supporting Information). With continuously extending the self‐assembly period, the nanopatches self‐arranged into a well‐defined structure (Figure S27C, Supporting Information) and eventually grew to the leaf‐like lamella within 1 h (Figure S27D, Supporting Information). It was believed that hydrophilic MeCN with high polarity enable DAdDMA@2*β*‐CD to stack tightly in MeCN/H_2_O mixture because of the high hydrophobicity of DAdDMA. These preliminarily formed sandwich‐like small aggregations were anisotropic in the lateral direction with the hydrophobic DAdDMA inside and hydrophilic *β*‐CD as the periphery, then driven by solvent effect and thermodynamics, these structures would hierarchically self‐assemble at two dimension direction and form large well‐defined leaf‐like lamella finally (Figure 4C). The same phenomenon was also displayed in DMF/H_2_O and ethanol/H_2_O systems. While, THF having lower polarity can afford better dissolution of hydrophobic DAdDMA moiety, and thus with increasing the THF faction in THF/H_2_O mixture, the building blocks can rearrange its molecular packing model, consequentially resulting in the morphology change of the self‐assembly. When the THF faction is 10–40%, DAdDMA@2*β*‐CD would preliminarily self‐assemble to small looser aggregations or helical aggregations, and then tend to grow in one dimension direction to form nanoribbons or helical nanofibers (Figure 4C). If further switching the ratio of THF/H_2_O to 1:1, DAdDMA@2*β*‐CD could stack into bended film‐like aggregations, and further grow into vesicle. It seems reasonable to infer that the temperature‐induced topological shape regulation is closely related with the greatly weakened multiple intermolecular interactions including hydrophobic interactions and hydrogen bonding, combining with the solvent polarity effect, DAdDMA@2*β*‐CD would stack at one dimension direction to form fiber‐like aggregations (Figure 4C) and hierarchically self‐assemble into the nanofibers. Furthermore, driven by solvent effect and surface tension, the nanofibers gradually bended and generated ring‐like structure,^[^ ^48^, ^49^, ^50^ ^]^ and the winding coil‐like multi‐ring structure can be constructed upon continuous increasing the THF content.

**Figure 3:**
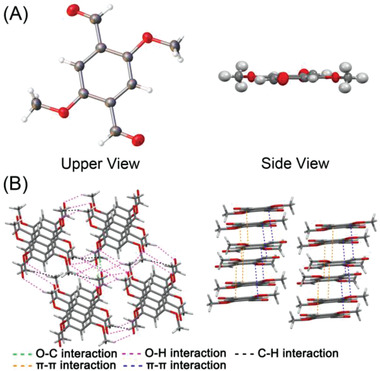
Single crystal data of DMA. A) Upper and side view of structure of DMA. B) The molecular packing model and intramolecular interaction of DMA.

**Figure 4:**
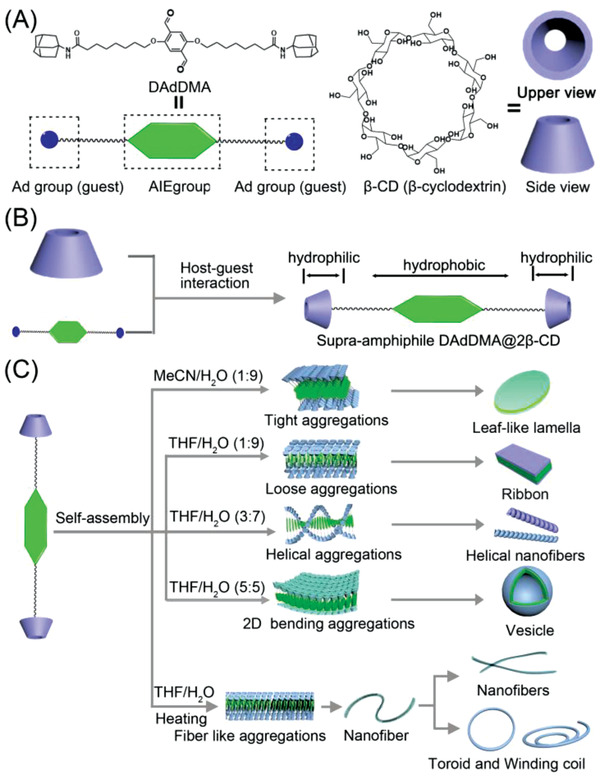
Schematic illustration of constructing diversiform topological morphologies using AIE supra‐amphiphiles. A) The structures of DAdDMA and *β*‐CD. B) Fabrication of supra‐amphiphile DAdDMA@2*β*‐CD. C) Achieving the formation of diversiform topological morphologies of self‐assembling DAdDMA@2*β*‐CD.

Luminescent property of these supramolecular topologies was further investigated by utilizing stimulated emission depletion (STED) nanoscopy. As shown in **Figure** **5**A,B and Figure S28 (Supporting Information), the fluorescence images of monodispersed and well‐defined leaf‐like lamella structures were clearly visualized, showing high signal‐to‐noise ratio, high fluorescence intensity and excellent photostability for both cases constructed in MeCN/H_2_O and ethanol/H_2_O systems. These notable features of leaf‐like lamella can be attributed to their AIE characteristics and the tight packing model under high polarity solvent system, remarkably indicating their potential application as fluorescent nanotemplate. In addition, relatively weak fluorescence emissions were determined in the nanoribbons (Figure 5C), nanofibers (Figure 5D), vesicles (Figure 5E), and toroids (Figure 5F) architectures, which were reasonably caused by the loose packing of these self‐assembled aggregates. Loose packing allows relatively active intramolecular motions, and consequently elevates energy dissipation from the pathway of thermal deactivation, resulting in inefficient fluorescence emission.

**Figure 5:**
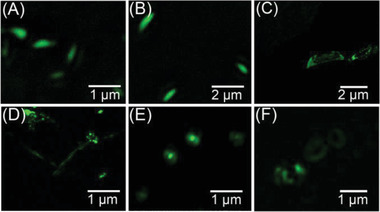
Fluorescence images of supramolecular topologies captured by STED nanoscopy. Fluorescence images of leaf‐like lamella constructed from self‐assembly of DAdDMA@2*β*‐CD in A) MeCN/H_2_O at 30 °C and B) ethanol/H_2_O at 30 °C. Fluorescence images of C) ribbons (THF/H_2_O = 1:9, 60 °C), D) nanofibers (THF/H_2_O = 3:7, 30 °C), E) vesicles (THF/H_2_O = 5:5, 30 °C), and F) nanotoroids (THF/H_2_O = (), 60 °C).

Light‐induced FRET process plays an important role in light‐harvesting organisms of green plants and some photosynthetic bacteria.^[^ ^51^, ^52^, ^53^ ^]^ Construction of high efficient light‐harvesting antenna system through FRET is an efficient way to mimic light harvesting system. Implementation of FRET requires some critical factors. The donor should be densely packed without significantly self‐quenching effect, and the distance of donor/acceptor should be no longer than 10 nm; moreover, the emission of donor and the absorption of acceptor must certainly have good overlap, which allows the transfer of fluorescence resonance energy among adjacent donors and benefits the energy transfer from donor to acceptor.^[^ ^54^, ^55^ ^]^ Several scaffolds for artificial light‐harvesting system have been exploited recently, such as protein arrays,^[^ ^56^, ^57^, ^58^ ^]^ DNA templates,^[^ ^59^, ^60^ ^]^ and other hybrid materials.^[^ ^61^, ^62^ ^]^ While, most of these artificial light‐harvesting systems suffer from the complex design and undesired aggregation‐caused quenching (ACQ) effect in water. The development of high‐performance artificial light‐harvesting antenna is still a fascinating target. Considering of the large specific surface and efficient emission of the presented self‐assembling DAdDMA@2*β*‐CD aggregates with leaf‐like lamellar morphology, they would be a wonderful donor for FRET platform. It was also demonstrated the fluorescence emission wavelength of DAdDMA ranging from 425 to 600 nm has good overlap with the absorption of Rhodamine B (RHB) ranging from 450 to 600 nm (**Figure** **6**A). In addition, owing to less than 2 nm of distance between *β*‐CD and DMA fragments (Figure 6B), if the *β*‐CD was modified with RHB, the distance between RHB and DMA would satisfy FRET requirement. Moreover, as RHB is a typical ACQ dye, modifying it on the *β*‐CD can also avoid the notorious ACQ effect during self‐assembly. Combination of these conditions, a highly effective mimicking light‐harvesting antenna system could be achieved on the basis of the novel supramolecular scaffolds (Figure 6C).

**Figure 6:**
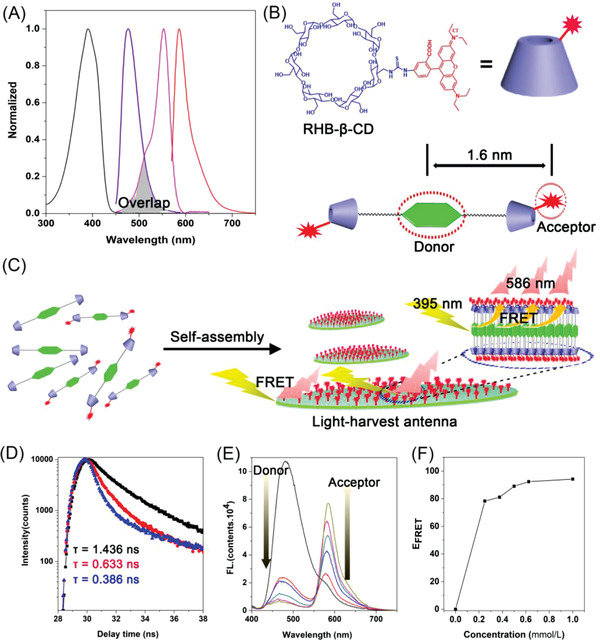
A) Normalized absorption spectra and fluorescence emission spectra of donor and acceptor (black profile is the UV spectrum of donor; purple profile is emission spectrum of donor; pink profile is the UV spectrum of acceptor; red profile is the emission spectrum of acceptor). B) Scheme of construction of RHB decorated supra‐amphilic (DAdDMA@2RHB‐*β*‐CD). C) Scheme of construction of light harvesting antenna by using RHB endowed leaf‐like lamella as scaffold. D) Donor fluorescence decay curves (black line: absence of DAdDMA@2RHB‐*β*‐CD; red line: 35% of DAdDMA@2RHB‐*β*‐CD; blue line: 100% of DAdDMA@2RHB‐*β*‐CD). The excitation wavelength of the sample is 395 nm. E) Fluorescence spectra of donor/acceptor in water with different concentration of DAdDMA@2RHB‐*β*‐CD. F) The FRET effect with different concentrations of DAdDMA@2RHB‐*β*‐CD.

In the preliminary study, RHB modified *β*‐CD (RHB‐*β*‐CD) was prepared by the reaction of RHB isothiocyanate (RHB‐SCN) with amino‐*β*‐cyclodextrin (Figure 6B; Scheme S2, Supporting Information). SEM image indicated that leaf‐like lamellar structure was smoothly constructed by employing RHB‐modified DAdDMA@2*β*‐CD supra‐amphiphiles under the same condition as unmodified DAdDMA@2*β*‐CD (Figure S29, Supporting Information). Confocal laser scanning microscope (CLSM) images revealed that the lamella consisting of unmodified DAdDMA@2*β*‐CD exhibit strong green fluorescence (Figure S30A, Supporting Information); with adding the DAdDMA@2*β*‐CD, the resulting self‐assembled micelles could be directly observed with red fluorescence, which showed good overlap with that of green signal (Figure S30B,C, Supporting Information), indicating that the energy was successfully transferred from DMA units encapsulated inside the lamella to the RHB groups on the surface through a light‐harvesting process.

Besides the fluorescent images, lifetime decay analysis was further used to evaluate the light‐harvesting process. The average lifetime of lamella consisting of unmodified DAdDMA@2*β*‐CD was 1.436 ns (black line in Figure 6D), but it changed to 0.633 ns after being decorated with RHB (the mole ratio of RHB‐*β*‐CD is 35%) (red line in Figure 6D). If the self‐assembling building blocks were completely RHB‐modified DAdDMA@2*β*‐CD, the lifetime of self‐assembled aggregates even reached to 0.386 ns (blue line in Figure 6D), indicating the efficient energy transfer from donor DMA units to acceptor RHB groups. Quantitative experiments were also performed to assess the light‐harvesting process. As shown in Figure 6E, with the addition of RHB‐*β*‐CD, the emission intensity at 586 nm belonging to the emission of RHB was enhanced and that at 466 nm (emission peak of donor) was gradually attenuated when excited at 395 nm. Moreover, the FRET efficiency (*E*_FRET_ = 1 − *I*_DA_/*I*_D_)^[^ ^61^ ^]^ was calculated, as illustrated in Figure 6F, the *E*_FRET_ was significantly magnified when the acceptor content was raised, and the maximal *E*_FRET_ reached to 94.2%, remarkably implying the high energy transfer efficiency from DMA to RHB in the self‐assembled lamella.

In summary, a novel bola‐type of supra‐amphiphiles DAdDMA@2*β*‐CD was successfully synthesized through noncovalent host–guest interaction between an AIE‐active guest and *β*‐CD host. The results described herein demonstrate that the supra‐amphiphiles can self‐assemble into well‐defined nanostructures, notably, multiple topological morphologies are able to be preciously regulated by simply tuning solvent proportioning and/or temperature, achieving the diversiform topological morphologies transforming from leaf‐like lamellar structure to nanoribbon, vesicle, nanofiber, helical nanofiber, and toroid. To the best of our knowledge, the presented supramolecular self‐assembly protocol holds the most types of morphology alterations based on a single entity comparing with those previously reported systems, and a few topological morphologies of self‐assembled nano‐aggregates are discovered for the first time for self‐assembly of AIE molecules. Mainly benefiting from the high‐performance fluorescence emission, the developed leaf‐like lamellas are successfully utilized mimicking light‐harvesting antenna system by incorporating the RHB as acceptor, offering up to 94.2% of energy transfer efficiency. The outcomes demonstrated in this study will cast a new light on the design, synthesis, and self‐assembly of supra‐amphilics with AIE features, and stimulate the development of various topological morphology‐depended functional nanomaterials for many applications ranging from biotechnology to stimuli‐responsive materials.

## Conflict of Interest

The authors declare no conflict of interest.

## Supporting information

Supporting Information
