# Supplementary material for: Controllable and Diversiform Topological Morphologies of Self‐Assembling Supra‐Amphiphiles with Aggregation‐Induced Emission Characteristics for Mimicking Light‐Harvesting Antenna
Source: Adv Sci (Weinh). 2020 Sep 23;7(20):2001909. doi: 10.1002/advs.202001909 (PMC7578885; doi:10.1002/advs.202001909)
Supplement: Supplementary file 1 — Supporting Information [file ADVS-7-2001909-s001.pdf]

© 2020 Wiley-VCH GmbH

ADVANCED SCIENCE
Open Access

Supporting Information

for *Adv. Sci.*, DOI: 10.1002/advs.202001909

Controllable and Diversiform Topological Morphologies Of Self-Assembling Supra-Amphiphiles with Aggregation-Induced Emission Characteristics for Mimicking Light-Harvesting Antenna

Shuang Fu, Xiang Su, Meng Li, Shanliang Song, Lei Wang, Dong Wang, and Ben Zhong Tang**

Supporting Information
©Wiley-VCH 2019
69451 Weinheim, Germany

Controllable and Diversiform Topological Morphologies of Self-Assembling Supra-Amphiphiles with Aggregation-Induced Emission Characteristics for Mimicking Light-Harvesting Antenna

Shuang Fu, Xiang Su, Meng Li, Shanliang Song, Lei Wang, Dong Wang,* and Ben Zhong Tang*

Materials and Methods

Materials: 6-Bromohexanoic acid, amino-β-Cyclodextrin, Amantadine, 2,5-dihydroxyterephthalaldehyde, Rhodamine B isothiocyanate and other reagents were purchased from J&K, Meryer, Titan. All the chemicals used as supplied without further purification. Tetrahydrofuran (THF), dichloromethane (CH_2Cl_2), chloroform ($CHCl_3$), Triethylamine (TEA) were further dried by distillation.

Characterization: ^{1}H and ^{13}C NMR spectra were recorded on a Bruker AVANCE Ⅲ 500MHZ spectrometer using $CDCl_3$ as internal reference. High-resolution mass spectra (HRMS) were obtained on a Finnigan MAT TSQ 7000 Mass Spectrometer operating in a MALDI-TOF mode. Quantum yield was determined by a Quanta-integrating sphere. Absorption spectra were measured on a Milton Ray Spectronic 3000 array spectrophotometer. Steady-state photoluminescence (PL) spectra were recorded on a Perkin Elmer LS 55 spectrometer and Edinburgh FS5 fluorescence spectrophotometer. Fluorescence images were collected on Stimulated Emission Depletion Nanoscopy (STED Nanoscopy) by TCS SP8 STED 3X. The materials morphology and structure were investigated using High Resolution Scanning Electron Microscope (FEI APREO S, Netherlands), Field Emission Transmission Electron Microscope-F200 (JEM-F200). Atomic Force Microscopy (AFM) measurements were performed on a NanoScope Multimode AFM (Veeco, USA) using the tapping mode with a SiN4 tip. Size analyses were implemented using a Zetasizer Nano ZSP (Malvern NanoZSP+MPT-2).

SEM samples preparing: Dropping the self-assembly solution on the surface of a freshly silicon wafer for a few seconds followed by drying in air.

AFM samples preparing: Immersing a freshly silicon wafer of self-assembly solution for a few seconds followed by drying in air.

TEM samples preparing: Dropping 10 μL of the self-assembly solution on the formvar carbon-coated copper grids. The sample drops were dried under air flow.

STED samples preparing: Dropping 500 μL of the self-assembly solution on the glass slide, then, covering with the 0.17 mm thickness of microscope cover glass.

Synthesis and Characterization

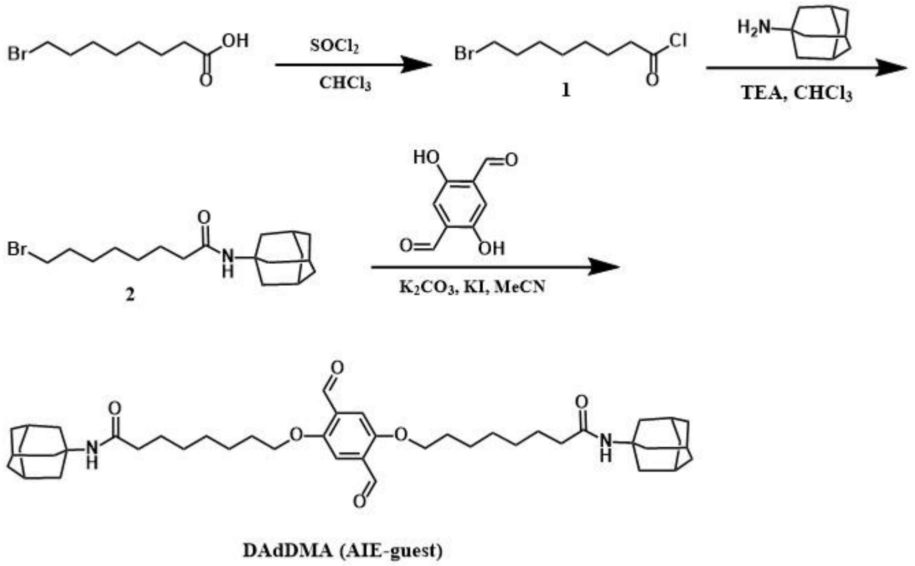

Scheme S1. Synthesis route towards DAdDMA.

Synthesis of N-(adamantan-1-yl)-8-bromooctanamide (2).

According to the scheme S1, in a 50mL of round bottom flask, 6-Bromohexanoic acid (2.22g, 0.01mol) was dissolved in 10 ml of anhydrous chloroform. Then an excess of $SOCl_2$ was dropped slowly in 30 min. The solution was stirred under nitrogen at room temperature for 20 h. Finally, the solvent and excess of $SOCl_2$ were removed under reduced pressure to yield the molecular 1. The molecular 1 was directly used to synthesize molecule 2. Molecular 1 was dissolved in 10 mL anhydrous chloroform ($CHCl_3$). Then, the Amantadine (2.5 g, 16.5mmol) was added, after then, 1 mL triethylamine (TEA) was dropped. The solution was stirred under room temperature for 18h. Then 50 mL H_2O was added and the crude product was extracted. After that, the combined extracts were dried over anhydrous $MgSO_4$, filtered, and the solvent removed under reduced pressure to yield a translucent oily solid. Purification was carried out via column chromatography. The ^{1}H-NMR (500MHz, Chloroform-d, 25℃, TMS) (Figure S1) δ: 3.41 (2H, H_1), 2.14 (2H, H_7), 2.09 (3H, H_9), 2.01 (6H, H_8), 1.87 (2H, H_2), 1.69 (6H, H_{10}), 1.62 (2H, H_6), 1.44 (2H, H_3), 1.34 (4H, H_4 and H_5). ESI-MS: calculated for C18H30NOBr, m/z 356.15; found, m/z 358.15 ($[M+H]^+$) (Figure S2).

Synthesis of DAdDMA

According to the scheme S1, 2,5-dihydroxyterephthalaldehyde (200 mg, 1.5mmol) was dissolved in acetonitrile (5 mL), then adding of molecular 2 (1.07 g, 3.3 mmol) and potassium carbonate (5g,). The resulting mixture was stirred at 60 ^{0}C for 27 h. After the reaction was cooled down to room temperature, solvent was removed under reduced pressure. The desired solid was purified with chromatography. The ^{1}H-NMR (500 MHz, Chloroform-d, 25℃, TMS) (Figure S3) δ: 10.5 (2H H_1), 7.42 (2H, H_2), 4.10 (4H, H_3), 2.18 (4H, H_9), 2.09 (6H, H_{11}), 2.02 (12H, H_{10}), 1.84 (4H, H_4), 1.69 (12H, H_{12}), 1.65 (4H, H_5), 1.49 (4H, H_8), 1.39 (8H, H_6 and H_7). The ^{13}C-NMR (500 MHz, Chloroform-d, 25℃, TMS) (Figure S4) δ: 189.00, 172.96, 155.00, 129.34, 111.63, 69.11, 52.31, 41.64, 37.63, 36.12, 29.43, 29.08, 29.04, 28.95, 28.00, 25.89, 25.84. ESI-MS: calculated for $C_{44}H_{64}N_2O_6$, m/z 717.48; found, m/z 718.48 ($[M+H]^+$) (Figure S5).

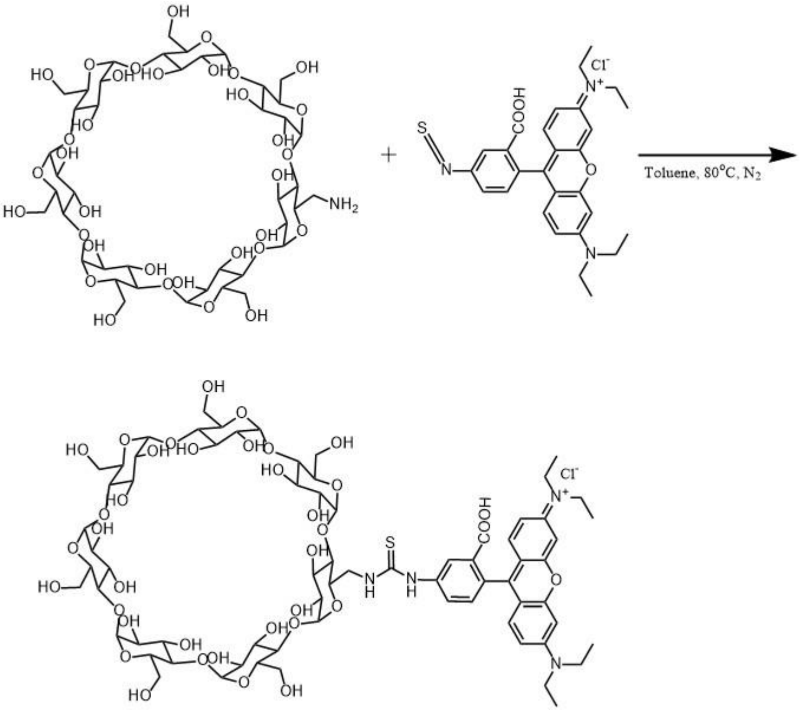

Scheme S2. Synthesis route towards RHB-β-CD.

According to the scheme S2,45.2 mg amino-β-Cyclodextrin (NH_2-β-CD) and 25.6 mg Rhodamine B isothiocyanate (RHB-SCN) were dissolved in 10mL toluene. The system was stirred at 80 ^{0}C for 12 h under nitrogen. After reaction, the solvent was removed to obtain the RHB-β-CD. ESI-MS: calculated for $C_{71}H_{101}N_4O_{37}S$, m/z 1633.59, found 1634.59 ($[M+H]^+$) (Figure S6).

NMR and HRMS spectra

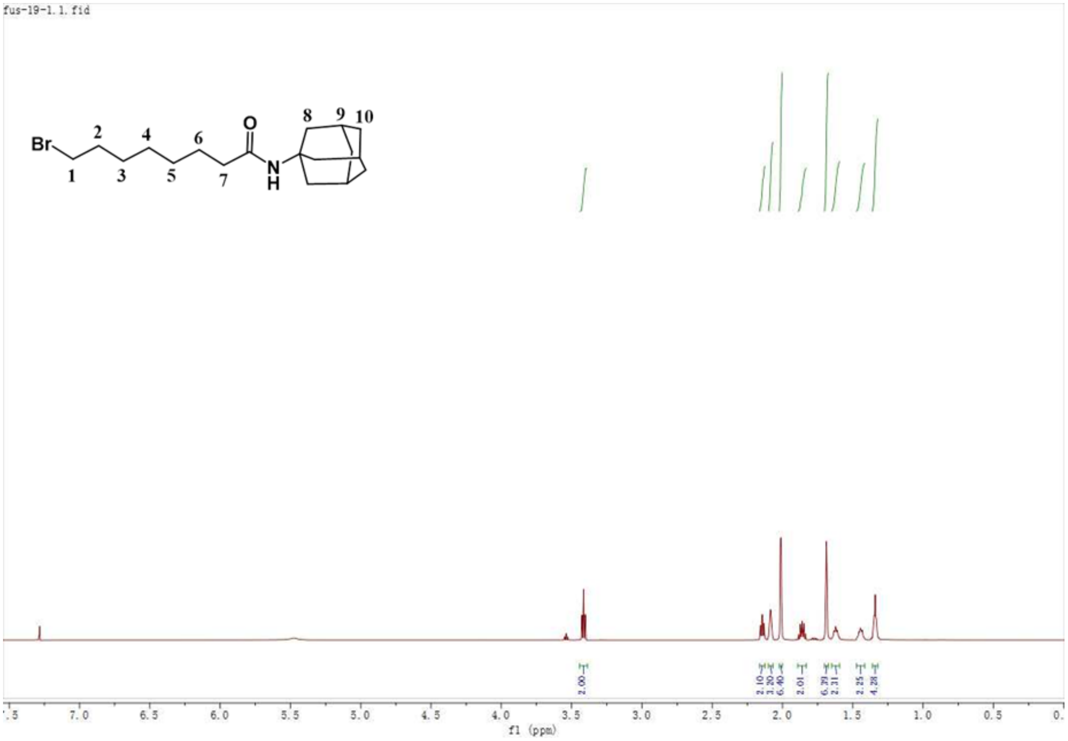

Figure S1. ^{1}H-NMR (500MHz, Chloroform-d, 25 ^{0}C, TMS) of N-(adamantan-1-yl)-8-bromooctanamide (2).

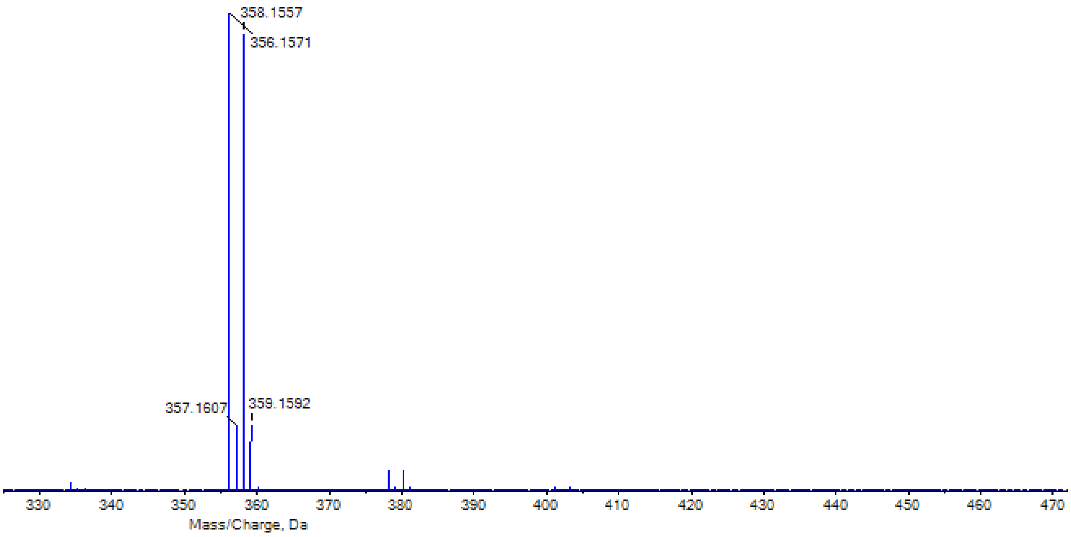

Figure S2. ESI-MS of 2 (calculated for $C_{18}H_{30}NO^{79}Br$, m/z 356.158 or $C_{18}H_{30}NO^{81}Br$, m/z 358.158). Found 356.157 and 358.1560 ($[M]^+$), 357.16 and 359.159 ($[M+H]^+$).

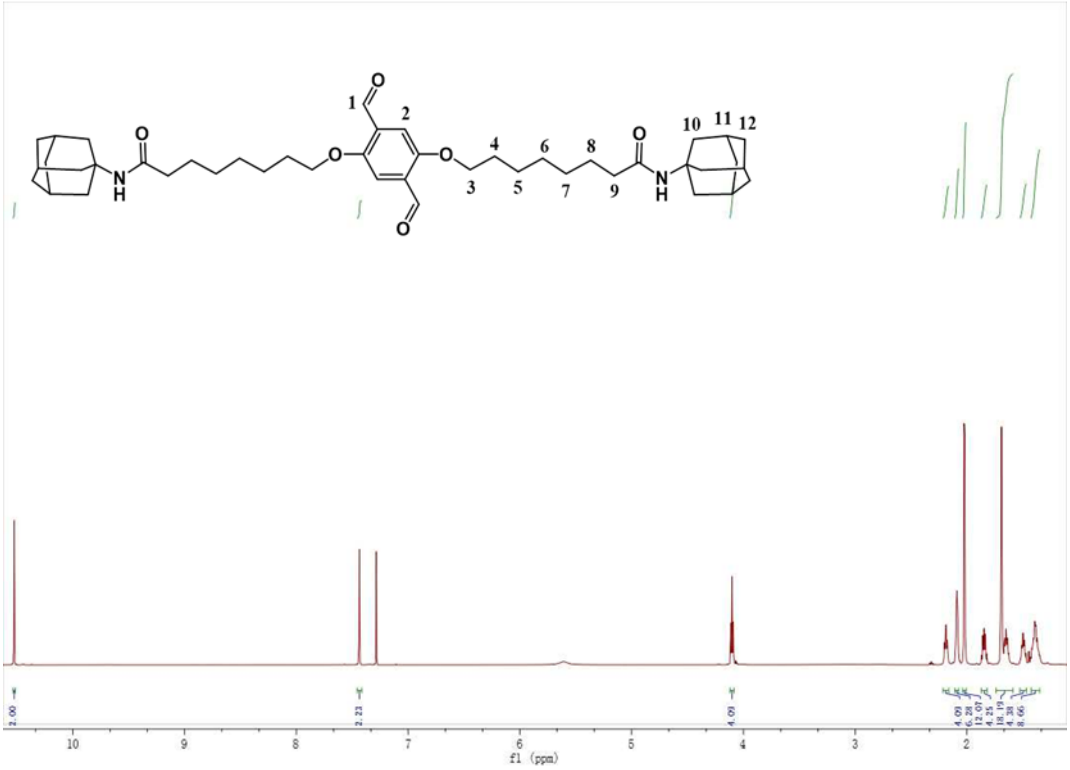

Figure S3. The ^{1}H-NMR (500MHz, Chloroform-d, 25 ℃, TMS) spectrum of DAdDMA.

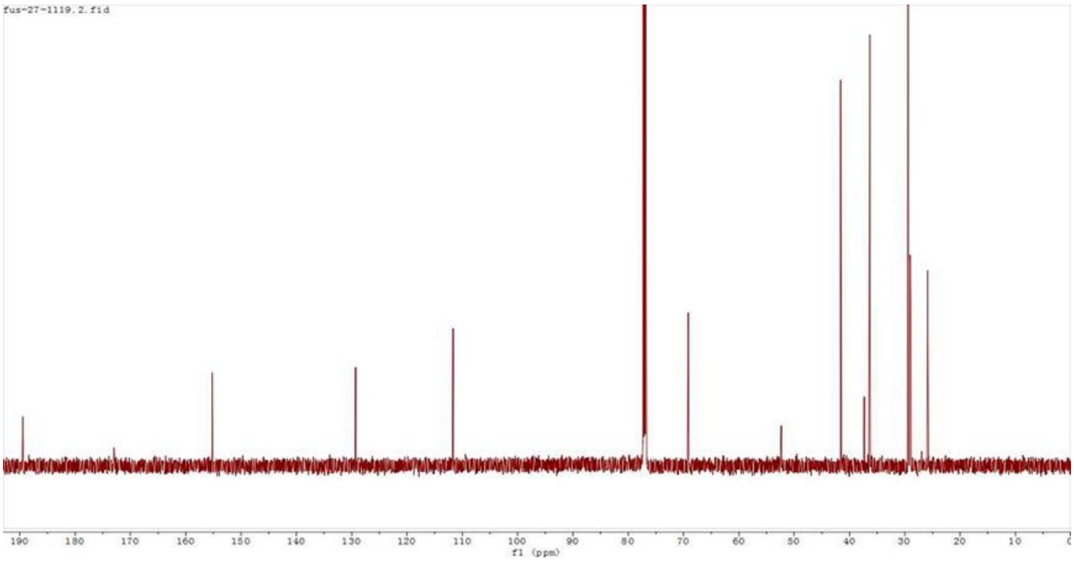

Figure S4. The ^{13}C-NMR (500MHz, Chloroform-d, 25℃, TMS) spectrum of DAdDMA.

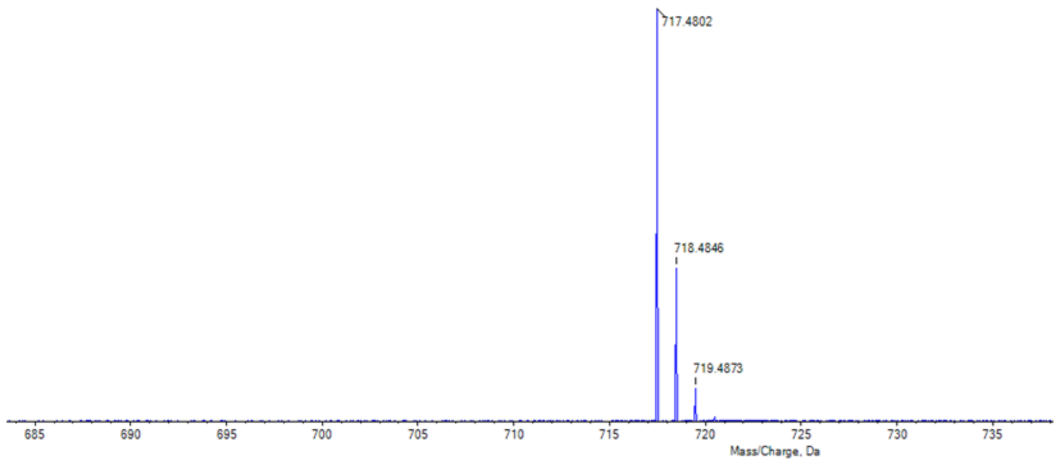

Figure S5. ESI-MS of DAdDMA. Calculated for $C_{44}H_{64}N_2O_6$, m/z 717.48; found, 717.48 ($[M]^+$), 718.48 ($[M+H]^+$).

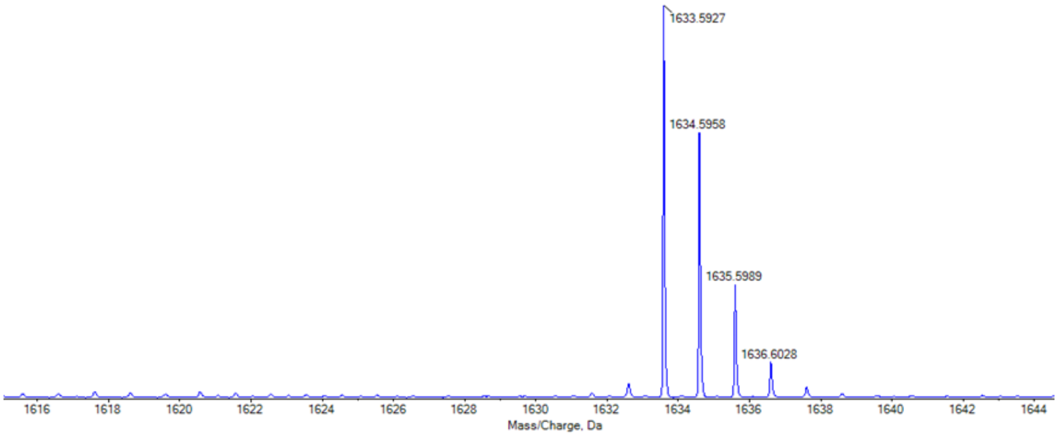

Figure S6. ESI-MS of RHB-β-CD. Calculated for $C_{71}H_{101}N_4O_{37}S$, m/z 1633.59, found 1634.59 ($[M+H]^+$).

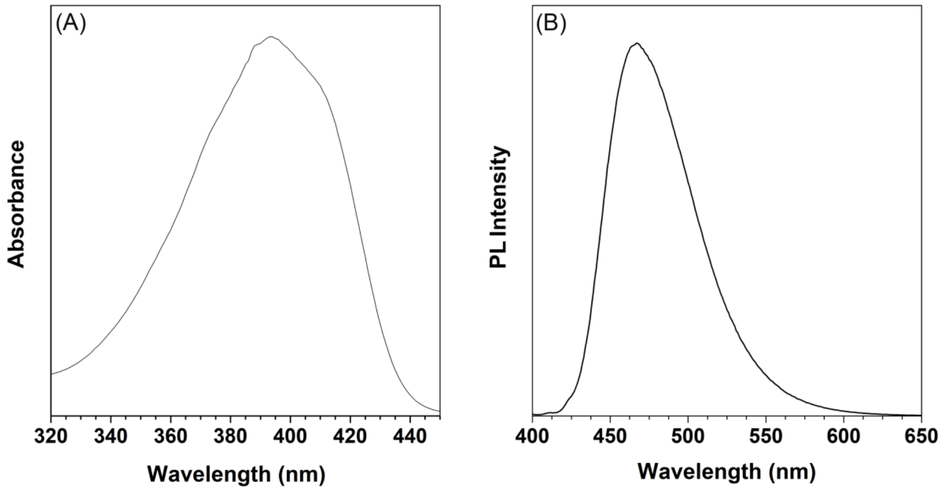

Figure S7. (A) Absorption and (B) fluorescence spectrum of DAdDMA in THF solution.

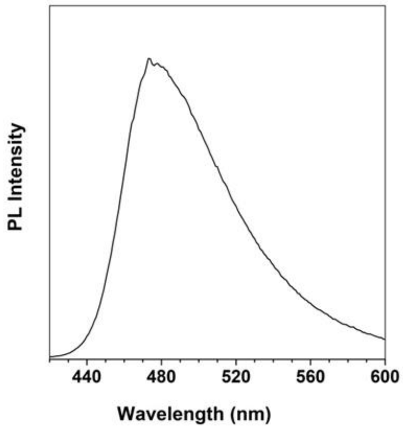

Figure S8. Fluorescence spectrum of DAdDMA in solid state.

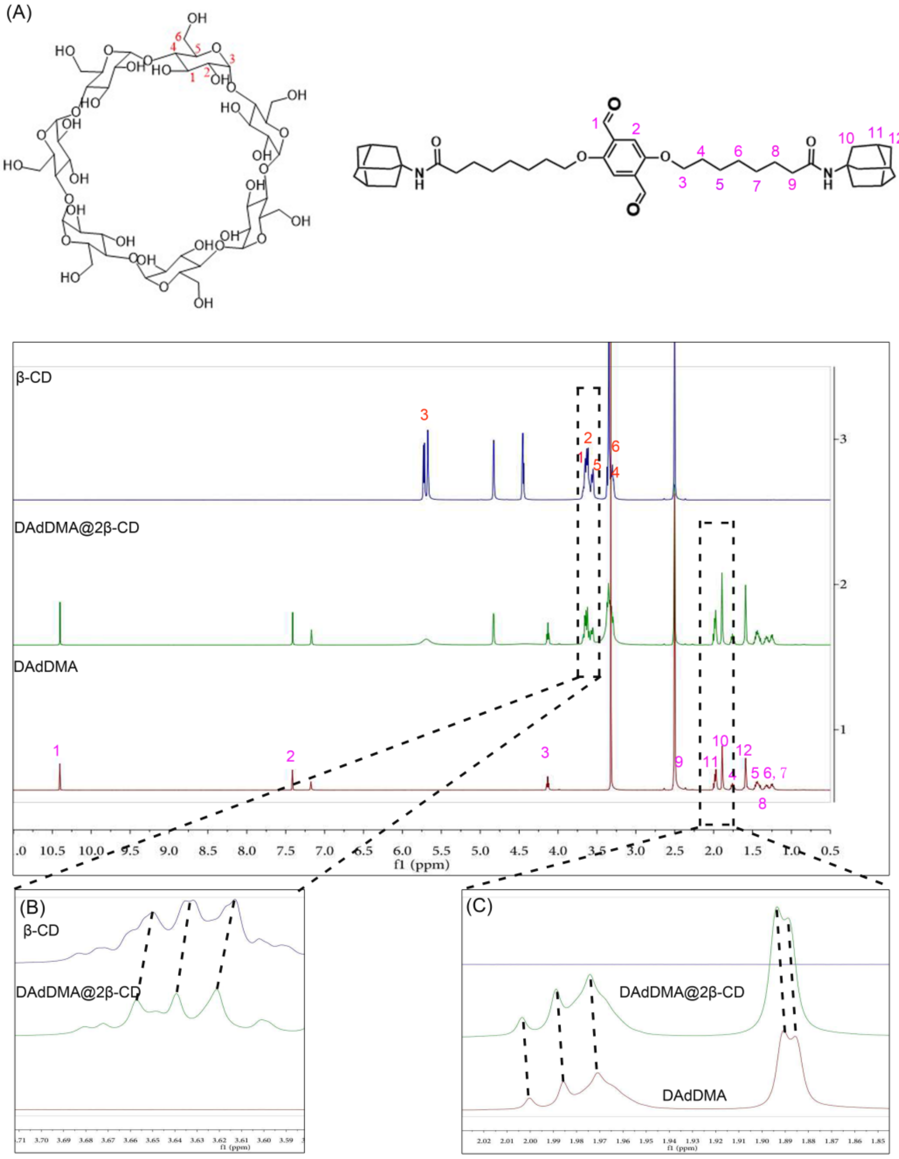

Figure S9. ^{1}H NMR spectra of β-CD, DAdDMA and hos-guest complex DAdDMA@2β-CD. (A) The general ^{1}H NMR spectra. (B) The amplified spectra of CD's proton of β-CD and hos-guest complex (DAdDMA:β-CD = 1:2). (C) The amplified spectra of Ad's proton of DAdDMA and host-guest complex DAdDMA@2β-CD.

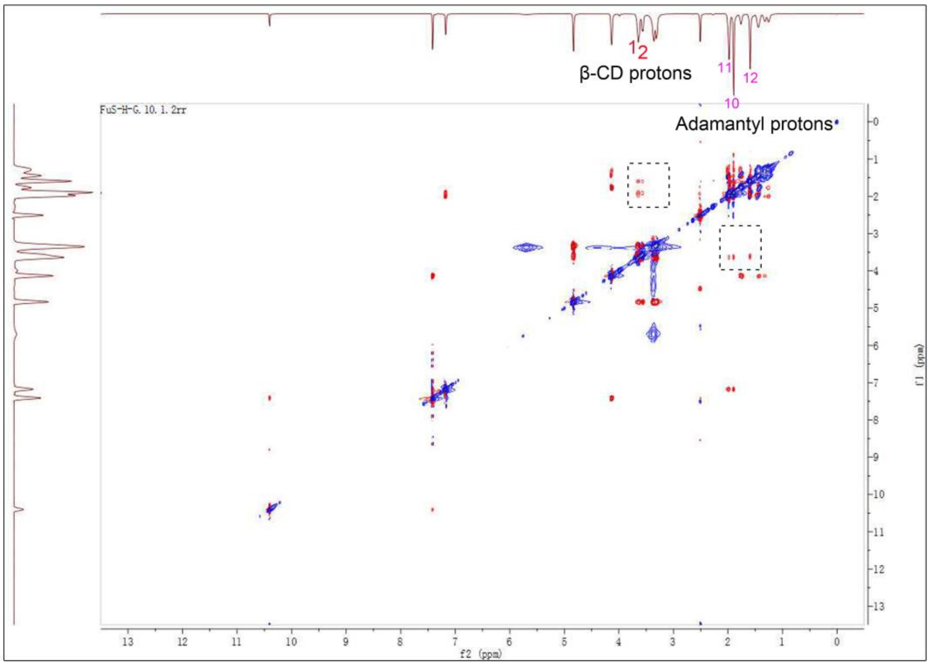

Figure S10. 2D NOESY ^{1}H NMR spectrum of a 1:2 mixture of the DAdDMA and the β-CD in DMSO-d_6 at 25 °C.

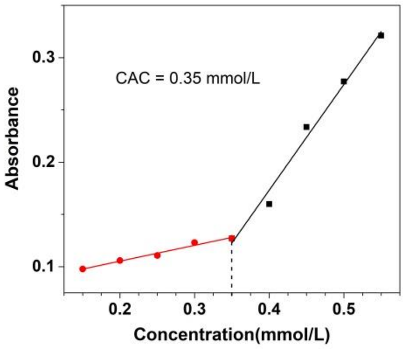

Figure S11. Dependence of absorption changes on DAdDMA@2β-CD concentration for critical aggregation concentration in MeCN/H_2O system at 30 °C.

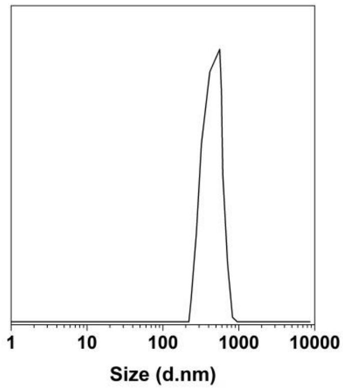

Figure S12. DLS analysis of leaf-like lamella for the self-assembly of DAdDMA@2β-CD in DMF/H_2O (1:9).

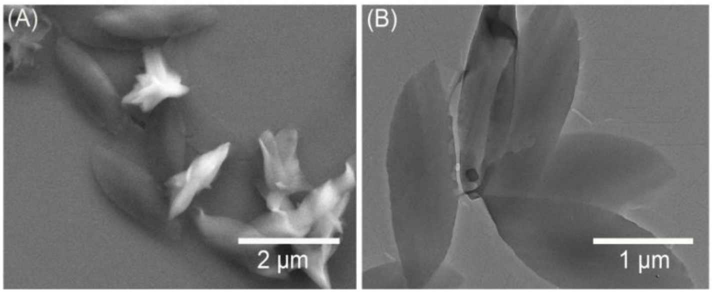

Figure S13. (A) SEM and (B) TEM images of leaf-like lamella for the supramolecular self-assembly of DAdDMA@2β-CD in EtOH/H_2O (1:9).

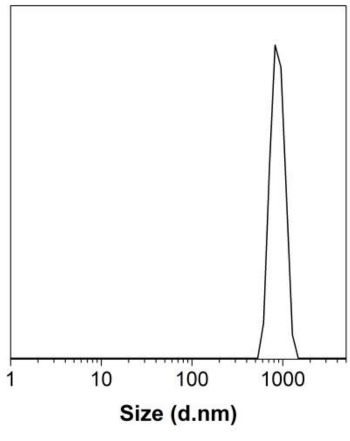

Figure S14. DLS analysis of leaf-like lamella for the supramolecular self-assembly of DAdDMA@2β-CD in EtOH/H_2O (1:9).

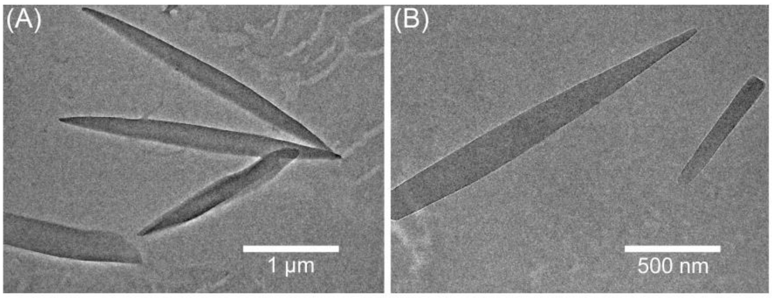

Figure S15. (A, B) TEM images of nanoribbons for supramolecular self-assembly of DAdDMA@2β-CD in THF/H_2O (1:9) at 30 °C.

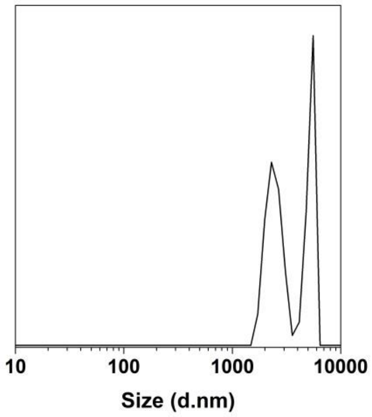

Figure S16. DLS analysis of nanoribbons for supramolecular self-assembly of DAdDMA@2β-CD in THF/H_2O (1:9) at 30 °C.

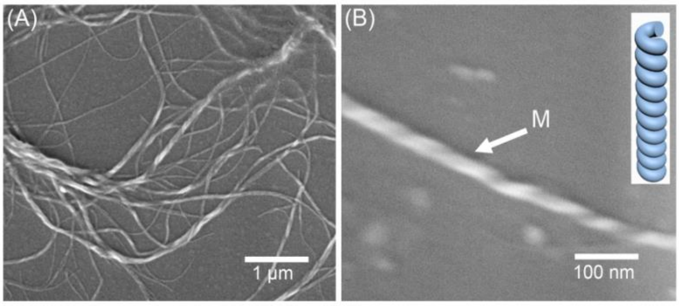

Figure S17. TEM images of helical nanofibers for supramolecular self-assembly of DAdDMA@2β-CD in THF/H_2O (3:7) at 30 °C. Inset: left handedness of the nanofiber.

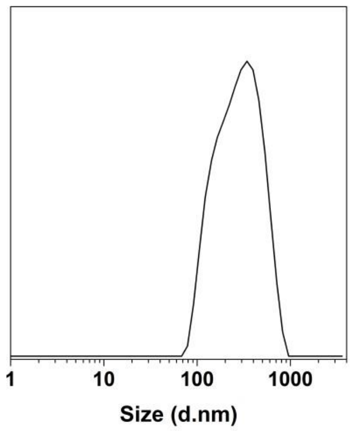

Figure S18. DLS analysis of helical nanofibers for self-assembly of DAdDMA@2β-CD in THF/H_2O (3:7) at 30 °C.

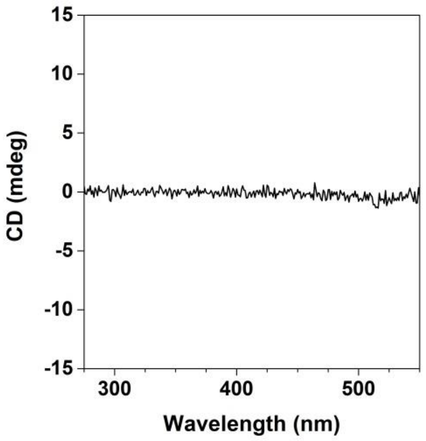

Figure S19. Circular dichroism (CD) analysis of helical fibers for self-assembly of DAdDMA@2β-CD in THF/H_2O (3:7) at 30 °C.

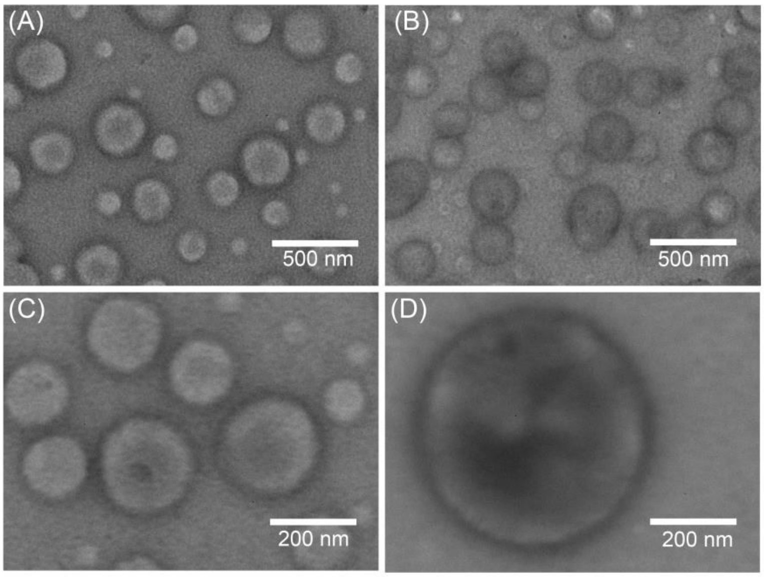

Figure S20. TEM images of vesicles for supramolecular self-assembly of DAdDMA@2β-CD in THF/H_2O (5:5) at 30 °C.

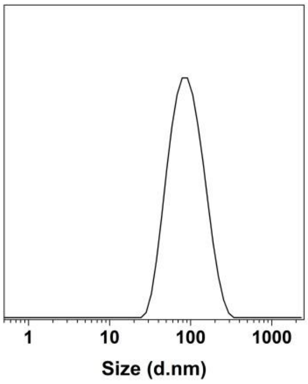

Figure S21. DLS anaylsis of vesicles for supramolecular self-assembly of DAdDMA@2β-CD in THF/H_2O (5:5) at 30°C.

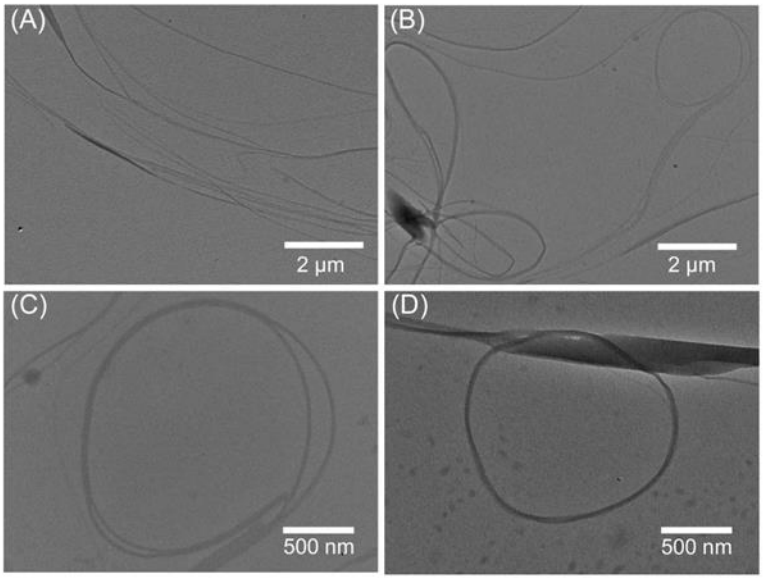

Figure S22. TEM images of nanotoroid structures for supramolecular self-assembly of DAdDMA@2β-CD in THF/H_2O (1:9) at 60 °C.

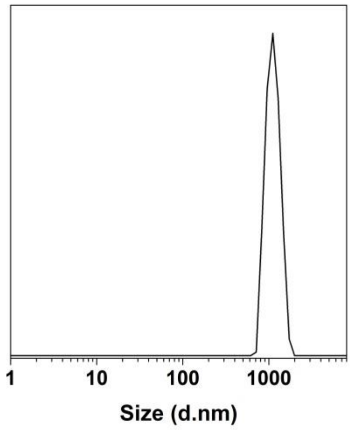

Figure S23. DLS anaylsis of nanotoroid structures for supramolecular self-assembly of DAdDMA@2β-CD in THF/H_2O (1:9) at 60 °C.

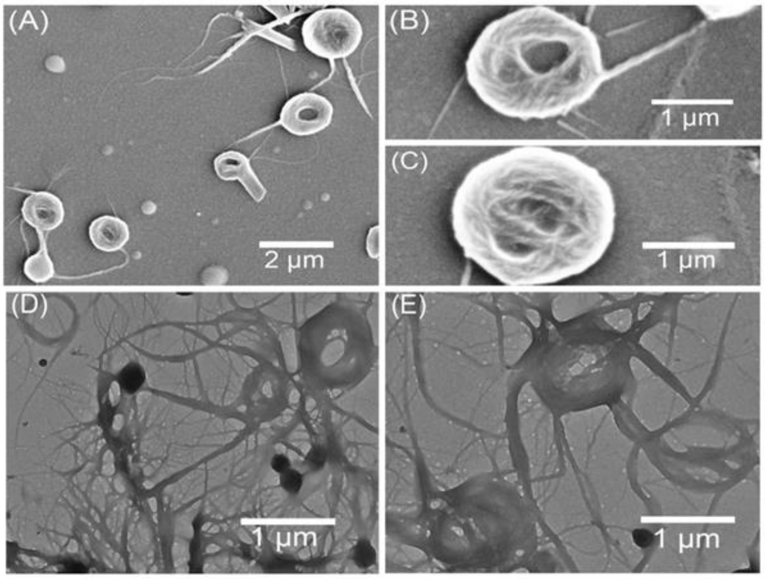

Figure S24. (A, B and C) SEM and (D, E) TEM images of winding coil structures. All the samples were from the supramolecular self-assembly of DAdDMA@2β-CD in THF/H_2O (4:6) at 60 °C.

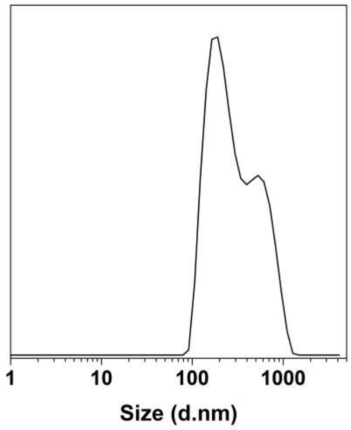

Figure S25. DLS anaylsis of winding coil structures for supramolecular self-assembly of DAdDMA@2β-CD in THF/H_2O (4:6) at 60 °C.

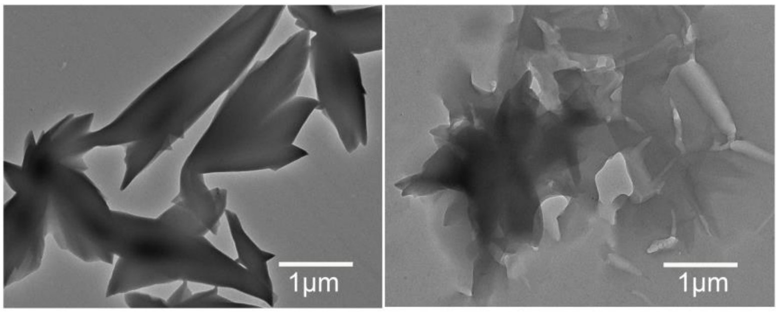

Figure S26. TEM images of morphological structures for supramolecular self-assembly of DAdDMA@2β-CD in MeCN/H_2O (1:9) at (A) 50 °C and (B) 60°C.

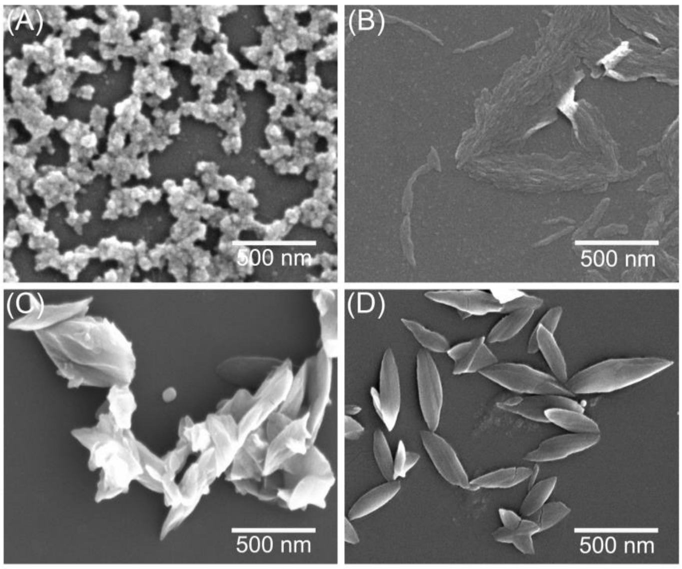

Figure S27. SEM images for monitoring the self-assembly process within (A) 5 min, (B) 15min, (C) 40 min and (D) 60 min. The experiment was conducted by taking the sample from the self-assembly system on time.

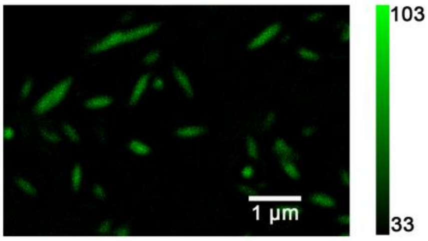

Figure S28. Fluorescence images of supramolecular topologies captured by STED nanoscopy. Fluorescence images of leaf-like lamella constructed from self-assembly of DAdDMA@2β-CD MeCN/H_2O (1:9).

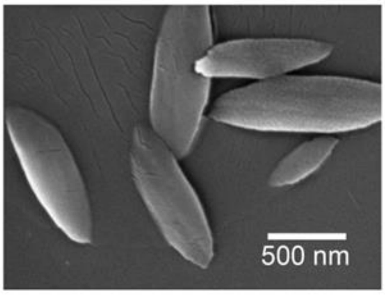

Figure S29. SEM image of leaf-like lamellar structure from self-assembly of RHB-decorated supra-amphilic (DAdDMA@2RHB-β-CD) in MeCN/H_2O (1:9) system at 30 °C.

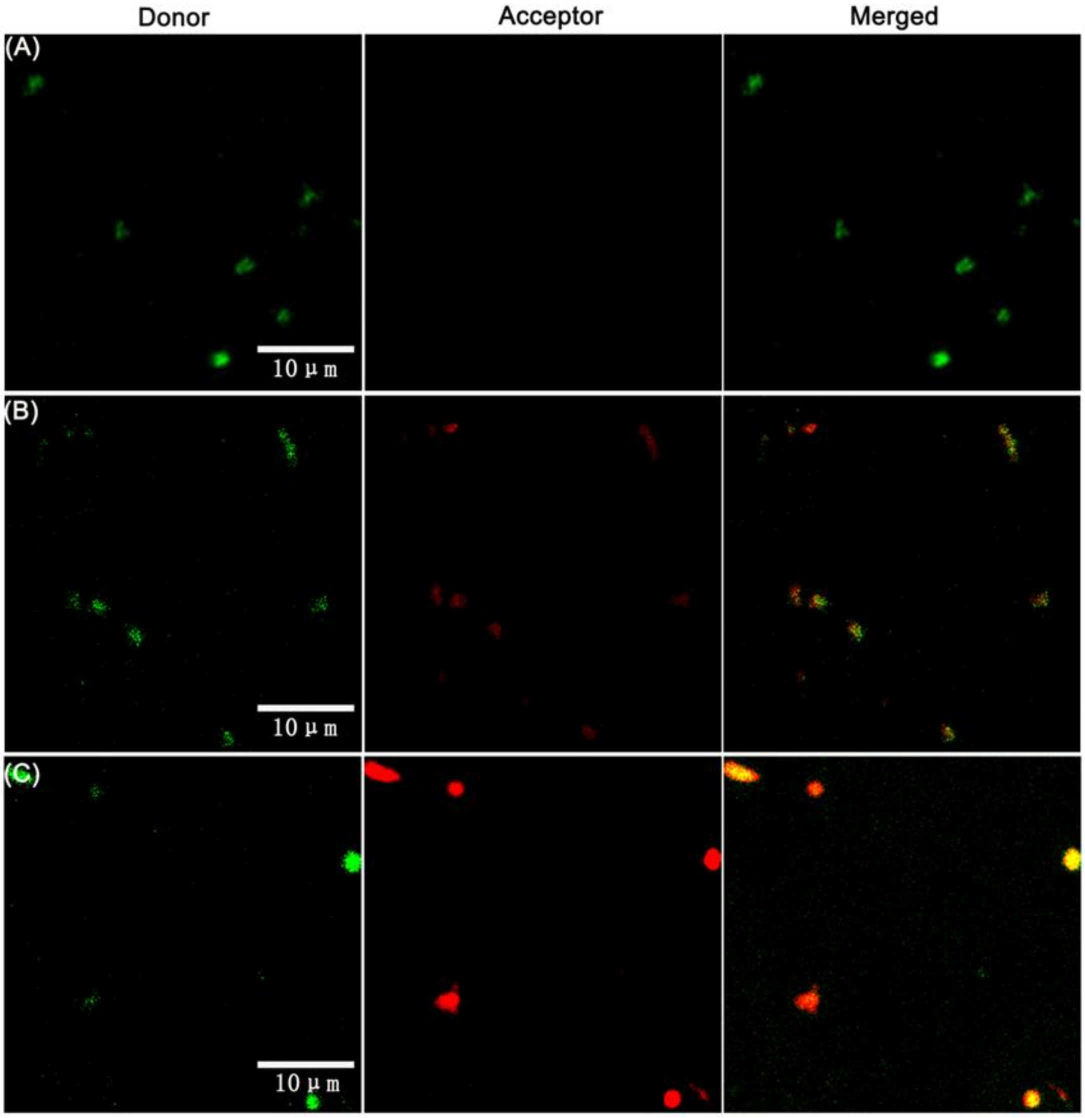

Figure S30. Confocal laser scanning microscope (CLSM) characterizations. (A) The free micelles without DAdDMA@2RHB-β-CD. (B) The lamellar structure with 35% DAdDMA@2RHB-β-CD. (C) The micelles with 100% DAdDMA@2RHB-β-CD.

Crystal data and structure analysis of DMA

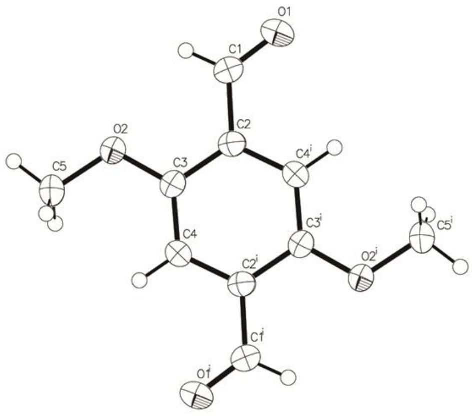

Table S1. Crystal data and structure refinement for DMA.

Identification code	**DMA**
Empirical formula	$C_{10}H_{10}O_4$
Formula weight	194.18
Temperature/K	293
Unit cell dimensions	a = 7.2861(12) Å α = 99.717°.
	b = 8.0474(9) Å β = 112.189°.
	c = 8.5158(10) Å γ = 93.590°.
Volume/ $Å^3$	451.32
$\rho_{calc}g/cm^3$	4, 1.429
Absorption coefficient/ mm^{-1}	0.111
F(000)	204
Crystal size/mm^3	0.35 x 0.19 x 0.17
2Θ range for data collection/°	3.720 to 29.365
Limiting indices	-9≤h≤9, -11≤k≤10, -10≤l≤11
Reflections collected	4604
Independent reflections	2067 [$R_{(int)}$ = 0.0319]

Completeness to theta = 25.242	99.1%
Max. and min. transmission	1.00000 and 0.06362
Refinement method	Full-matrix least-squares on F^2
Data / restraints / parameters	2067 / 0 / 129
Goodness-of-fit on F^2	1.109
Final R indices [I>=2σ (I)]	R1 = 0.0734, wR2 = 0.1667
R indices (all data)	R1 = 0.0975, wR2 = 0.1966
Extinction coefficient	n/a
Largest diff. peak/hole / e $Å^{-3}$	0.402 /-0.632

Table S2. Atomic coordinates ($x10^4$) and equivalent isotropic displacement parameters ($Å^2 \times 10^3$) for DMA. U (eq) is defined as 1/3 of the trace of the orthogonalized UIJ tensor.

**Atom**	***x***	***y***	***z***	**U(eq)**
O(4)	389(2)	1771(1)	-2396(1)	45(1)
O(2)	4294(2)	8573(1)	6601(1)	45(1)
O(1)	6061(2)	13560(2)	8157(2)	55(1)
O(3)	1439(2)	3173(2)	4038(2)	66(1)
C(3)	4619(2)	9217(2)	8284(2)	32(1)
C(4)	4382(2)	8258(2)	9413(2)	32(1)
C(7)	464(2)	798(2)	1708(2)	33(1)
C(2)	5253(2)	10980(2)	8881(2)	30(1)
C(8)	669(2)	1740(2)	546(2)	34(1)
C(1)	5540(3)	12038(2)	7725(2)	40(1)
C(9)	219(2)	962(2)	-1161(2)	33(1)
C(5)	3710(3)	6768(2)	5981(2)	50(1)

C(10)	996(3)	3562(2)	-1918(2)	49(1)
C(6)	975(3)	1669(2)	3511(2)	48(1)

Table S3. Bond Lengths for DMA.

**Atom**	**Atom**	**Atom**	**Length/Å**
O(4)	C(9)		1.365(2)
O(4)	C(10)		1.423(2)
O(2)	C(3)		1.3628(18)
O(2)	C(5)		1.4361(19)
O(1)	C(1)		1.210(2)
O(3)	C(6)		1.196(2)
C(3)	C(4)		1.381(2)
C(3)	C(2)		1.408(2)
C(4)	C(2)		1.395(2)
C(7)	C(8)		1.388(2)
C(7)	C(9)^{i}		1.409(2)
C(7)	C(6)		1.470(2)
C(2)	C(1)		1.467(2)
C(8)	C(9)		1.385(2)
C(9)	O(4)	C(10)	117.69(13)
C(3)	O(2)	C(5)	117.36(13)
O(2)	C(3)	C(4)	124.42(14)
O(2)	C(3)	C(2)	116.42(14)
C(4)	C(3)	C(2)	119.16(15)

C(3)	C(4) C(2)	120.76(14)
C(8)	C(7) C(9)^{i}	120.29(15)
C(8)	C(7) C(6)	118.86(15)
C(9)^{i}	C(7) C(6)	120.85(16)
C(3)	C(2) C(1)	120.76(15)
C(4)	C(2) C(3)	120.08(15)
C(4)	C(2) C(1)	119.17(14)
C(9)	C(8) C(7)	120.56(15)
O(1)	C(1) C(2)	123.77(16)
O(4)	C(9) C(7)^{i}	115.83(14)
O(4)	C(9) C(8)	125.03(14)
C(8)	C(9) C(7)i	119.14(16)
O(3)	C(6) C(7)	124.44(18)

Table S4. Anisotropic Displacement Parameters (Å^2×10^3) for DMA. The Anisotropic displacement factor exponent takes the form: $-2\pi^2[h^2a^{*2}U_{11}+2hka^*b^*U_{12}+\ldots]$.

Atom	U11	U22	U33	U23	U13	U12
O(4)	68(1)	32(1)	34(1)	8(1)	21(1)	-1(1)
O(2)	76(1)	30(1)	29(1)	2(1)	23(1)	-3(1)
O(1)	85(1)	33(1)	48(1)	13(1)	29(1)	-2(1)
O(3)	105(1)	40(1)	46(1)	-11(1)	35(1)	-10(1)
C(3)	38(1)	31(1)	26(1)	4(1)	14(1)	4(1)
C(4)	40(1)	25(1)	32(1)	5(1)	14(1)	1(1)
C(7)	35(1)	31(1)	31(1)	2(1)	13(1)	4(1)
C(2)	34(1)	30(1)	29(1)	9(1)	14(1)	4(1)

C(8)	41(1)	24(1)	36(1)	3(1)	16(1)	3(1)
C(1)	56(1)	36(1)	31(1)	9(1)	18(1)	2(1)
C(9)	39(1)	29(1)	33(1)	7(1)	16(1)	6(1)
C(5)	81(1)	32(1)	35(1)	-2(1)	26(1)	-4(1)
C(10)	70(1)	32(1)	47(1)	10(1)	26(1)	0(1)
C(6)	67(1)	40(1)	36(1)	1(1)	23(1)	-3(1)

Table S5. Hydrogen Atom Coordinates (Å×10^4) and Isotropic Displacement Parameters (Å^2×10^3) for DMA.

**Atom**	***x***	***y***	***z***	**U(eq)**
H(4)	3974	7091	9030	39
H(8)	1113	2903	916	41
H(1)	5308	11509	6596	49
H(5A)	3566	6468	4797	75
H(5B)	2459	6448	6055	75
H(5C)	4717	6182	6678	75
H(10A)	1082	3969	-2888	73
H(10B)	2281	3814	-967	73
H(10C)	34	4112	-1576	73
H(6)	937	1005	4296	58
